# The role of corticosteroids in severe community-acquired pneumonia: a systematic review

**DOI:** 10.1186/cc6922

**Published:** 2008-06-11

**Authors:** Jorge IF Salluh, Pedro Póvoa, Márcio Soares, Hugo C Castro-Faria-Neto, Fernando A Bozza, Patrícia T Bozza

**Affiliations:** 1 Intensive Care Unit, Instituto Nacional de Câncer, Rio de Janeiro, Brazil, Praça Cruz Vermelha, 23, 10 andar – Centro, 20230-130 – Rio de Janeiro – RJ; 2 Immunopharmacology Laboratory, Instituto Oswaldo Cruz, FIOCRUZ, Av. Brasil, 4365, Rio de Janeiro-RJ, Brazil 21045-900; 3 Medical Intensive Care Unit, Hospital de São Francisco Xavier. Centro Hospitalar de Lisboa Ocidental. Estrada do Forte do Alto do Duque, CEP 1449-005 Lisboa, Portugal; 4 Instituto de Pesquisa Clínica Evandro Chagas, Fundação Oswaldo Cruz, Av. Brasil, 4365, Rio de Janeiro-RJ, Brazil 21045-900

## Abstract

**Introduction:**

The purpose of this review was to evaluate the impact of corticosteroids on the outcomes of patients with severe community-acquired pneumonia (CAP).

**Methods:**

We performed a systematic MEDLINE, Cochrane database, and CINAHL search (1966 to November 2007) to identify full-text publications that evaluated the use of corticosteroids in CAP.

**Results:**

An initial literature search yielded 109 articles, and 105 studies were excluded after the first analysis. We found four studies eligible for analysis. On the basis of their results, the use of corticosteroids as adjunctive therapy in severe CAP should be categorized as a weak recommendation (two studies) and a strong recommendation (two studies) with either low- or moderate-quality evidence. However, no evidence of adverse outcomes or harm is present in the evaluated studies.

**Conclusion:**

According to the GRADE system, available studies do not support the recommendation of corticosteroids as a standard of care for patients with severe CAP. Further randomized controlled trials with this aim should enroll a larger number of severely ill patients. However, in patients needing corticosteroids, it may be reasonable to conclude that corticosteroid administration is safe in patients with severe infections receiving antimicrobial therapy.

## Introduction

Community-acquired pneumonia (CAP) is associated with significant morbidity and mortality and is the most common cause of death from infectious diseases in developed countries [1]. Patients with severe CAP often require intensive care unit admission and mechanical ventilation. An exceedingly high mortality rate is observed in these patients despite significant advances in etiological investigation, antimicrobial therapy, and improvements in supportive care [2]. With perhaps a few exceptions [3,4], adjunctive therapies for severe infections have failed to fulfill their promise in the past years [5].

Nevertheless, with the concept of critical illness-related corticosteroid insufficiency (CIRCI) and the results of clinical trials showing respiratory [6,7], immune [8,9], and hemodynamic [7] benefits, corticosteroids have re-emerged as promising adjuncts for the treatment of severe sepsis [10]. In the past years, several studies evaluated the pulmonary [11] and systemic [12] inflammatory response as well as data on the adrenal function [13,14] of patients with severe CAP. In addition, the administration of systemic corticosteroids is associated with reduced pulmonary inflammation in patients with bacterial pneumonia [11] and acute lung injury [15] and improved oxygenation and outcomes in patients with *Pneumocystis jirovecii *pneumonia [16]. Recent guidelines for the management of CAP suggest the benefit of systemic corticosteroids for patients with a severe presentation [1]. Yet at the time of their publication, only data from a single small randomized controlled trial (RCT) demonstrating improved survival were available [6]. At present, few studies have addressed the use of corticosteroids in the treatment of patients with CAP and their role is still unclear in this setting. In the present article, we reviewed recent peer-reviewed reports to determine whether systemic corticosteroids have an impact on the outcomes of patients with severe CAP. In addition, we explored possible explanations for the observed results.

## Materials and methods

### Selection of participants, data collection, and definitions

We performed a systematic search of MEDLINE, the Cochrane database, and CINAHL (for the period of 1966 to November 2007) to identify full-text English language publications that evaluated the use of corticosteroids in CAP. Inclusion criteria were established *a priori*. Major MeSH (Medical Subject Heading) search terms included 'community-acquired infections', 'pneumonia', 'steroids', 'corticosteroids', 'glucocorticoids', and 'cortisol'. We also reviewed the references of available studies for other potentially eligible studies, and additional published reports were identified through a manual search of citations from retrieved articles. Only original peer-reviewed clinical trials and cohort studies evaluating the use of corticosteroids in patients with severe CAP were selected and analyzed. The abstracts of all articles were used to confirm our target population (patients with CAP), and the corresponding full-text articles were reviewed for the presence of data evaluating the outcomes of adult non-immunocompromised patients with severe CAP treated with systemic corticosteroids. Only studies of at least 20 adult patients were included. We excluded case reports, articles in which children were the subject of study, articles on CAP in HIV-infected patients, nosocomial pneumonia, or CAP managed in an outpatient setting. Two investigators (JIFS and PP) independently identified the eligible literature. Predefined variables were collected, including year of publication, study design (prospective/retrospective), number of patients included, and hospital mortality and length of stay (LOS), oxygenation, markers of systemic inflammation, and data on pneumonia severity. Additional unpublished data were obtained by electronic mail from Garcia-Vidal and Mikami. Any inconsistencies between the two investigators (JIFS and PP) in interpretation of data were resolved by consensus. The data were extracted to a standardized form and included the number of patients, hospital mortality and LOS, oxygenation, markers of systemic inflammation, and data on pneumonia severity. In addition, we explored possible reasons that may explain the results and assigned quality levels as defined by the American Thoracic Society (ATS) GRADE system to formulate the level of recommendation [17].

## Results

**Figure 1:**
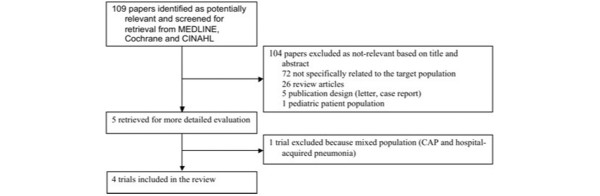
Flow diagram of selection of studies. CAP, community-acquired pneumonia.

The initial literature search yielded 109 articles. One hundred four studies were excluded based on their titles and abstracts. One article was excluded after revision for involving both patients with CAP and hospital-acquired pneumonia [18]. The complete flow diagram and reasons for exclusion are presented in Figure 1. Eventually, we selected four studies that evaluated the use of corticosteroids for severe CAP. These studies are summarized in Tables 1 and 2.

**Table 1:** Clinical studies on the role of corticosteroids in severe community-acquired pneumonia

Reference	Study design	Sample size, number	Patient selection	Corticosteroids (drug/regimen)	Primary endpoints	Level of evidence and recommendation
Marik, *et al*. [21]	Single-center RCT	30	Severe CAP	Hydrocortisone 10 mg/kg versus placebo 30 minutes before antibiotics	Mortality, clinical course, and serum TNF-α levels	Weak recommendation, moderate-quality evidence
Confalonieri, *et al*. [6]	Multicenter RCT	46	Severe CAP	Hydrocortisone 200 mg + hydrocortisone 10 mg/hour for 7 days versus placebo	Mortality, clinical course, and systemic inflammation	Strong recommendation, moderate-quality evidence
Mikami, *et al*. [20]	Open-label RCT	31	Moderate and severe CAP	Prednisolone 40 mg qd for 3 days versus placebo	Mortality and clinical course	Weak recommendation, low-quality evidence
Garcia-Vidal, *et al*. [19]	Retrospective cohort study	308	Severe CAP	Methylprednisolone 14.5 mg (or equivalent) qd for 11.4 days	Mortality	Strong recommendation, low-quality evidence

CAP, community-acquired pneumonia; qd, *quaque die *(every day); RCT, randomized controlled trial; TNF-α, tumor necrosis factor-alpha.

**Table 2:** Effects of corticosteroids versus placebo on different endpoints of severe community-acquired pneumonia studies

Reference	Mortality, percentage	Clinical course			Systemic inflammation
		LOS, days	MODS	AB duration, days	
Marik, *et al*. [21]	7.1 versus 18.8 (*P *= NS)	4.6 versus 4.3	N/A	N/A	Plasma TNF-α concentrations; no effect
Confalonieri, *et al*. [6]	0 versus 30 (*P *= 0.009)	10 versus 18 (*P *= 0.01)	0.3 versus 1.0 (*P *= 0.003)	N/A	Lower CRP levels at day 8 18 versus 34 mg/dL (*P *= 0.01)
Mikami, *et al*. [20]	6 versus 0 (*P *= 0.99)	11.3 versus 15.5 (*P *= 0.182)	N/A	8.5 versus 12.3 (*P *= 0.026)	CRP Faster normalization of CRP levels (7.6 versus 11.7 days; *P *= 0.02)
Garcia-Vidal, *et al*. [19]	OR, 0.287; 95% CI, 0.113 to 0.732	7.1 versus 13.8 (*P *= 0.005)	N/A	N/A	N/A

AB, antibiotic therapy; CI, confidence interval; CRP, C-reactive protein; LOS, length of stay; MODS, multiple organ dysfunction score; N/A, not available; NS, not significant; OR, odds ratio; TNF-α, tumor necrosis factor-alpha.

Two studies found a significant reduction in mortality for patients with severe CAP treated with corticosteroids [6,19]. The study of Confalonieri and colleagues [6], a small-sized (n = 46) multicenter RCT, showed an impressive reduction in hospital mortality (30% versus 0%; *P *= 0.009) with a 7-day continuous infusion of hydrocortisone (240 mg/day). Garcia-Vidal and colleagues [19] evaluated 308 patients in a large retrospective single-center cohort study and demonstrated in a multivariate analysis that the use of corticosteroids was strongly associated with a lower mortality (hospital mortality; odds ratio, 0.287; 95% confidence interval, 0.113 to 0.732). These patients were treated with a median dose of 45 mg/day of methylprednisolone for a mean period of 11.4 days. In a small open-label RCT evaluating 31 patients hospitalized for CAP, no mortality benefit was observed in patients receiving a 3-day course of 40 mg of prednisolone as compared with placebo [20]. Marik and colleagues [21] evaluated the effects on clinical course of a single dose of hydrocortisone administered to patients (n = 30) with severe CAP 30 minutes before antibiotic therapy. In this small RCT, no improvement in survival was observed.

Regarding the LOS, two studies found a reduction in LOS for patients treated with corticosteroids [6,19] which could not be demonstrated in the remaining studies [20,21]. The role of CIRCI was not systematically addressed in any of the studies. Only the study of Mikami and colleagues [20] evaluated the adrenal function of patients with CAP. In a subset of patients (23/31), total cortisol levels at baseline and after ACTH (adrenocorticotropic hormone) stimulation were measured showing a 43% prevalence of CIRCI. Only the study by Confalonieri and colleagues [6] provides detailed data on the presence of acute lung injury and septic shock, only. In the study by Confalonieri and colleagues [6], 34 (79%) of all patients had arterial partial pressure of oxygen/fraction of inspired oxygen (PaO_2_/FiO_2_) ratios of less than 200 mm Hg and most of them were in the placebo arm of the study (21 [91%] versus 13 [57%]; *P *= 0.01). Regarding the presence of shock at baseline, Confalonieri and colleagues [6] reported a low overall rate of vasopressor dependency (n = 3 [7%]).

Possible side effects of using corticosteroids, such as increased risk of nosocomial infections, myopathy, and hyperglycemia, were not systematically investigated and are not reported by most studies [19-21]. However, no increased hospital LOS or mortality was observed. Moreover, Confalonieri and colleagues [6] prospectively evaluated all patients enrolled in the study for the occurrence of such complications and could not demonstrate an increased risk of such complications in patients using hydrocortisone as compared with placebo.

### Design and patient selection

Three studies were designed as a prospective RCT [6,20,21] and only one study evaluated a retrospective cohort [19]. Selection criteria were variable in the four studies. Confalonieri and colleagues [6] used the modified ATS criteria for severe CAP [22], and Marik and colleagues [21] applied the British Thoracic Society criteria for severe CAP [23]; both studies included a homogenous group of patients with severe CAP. Garcia-Vidal and colleagues [19] retrospectively evaluated only patients with severe CAP (classes IV and V of the pneumonia severity index [PSI] score) [24], and data were acquired from the medical charts. Despite the retrospective design, strict inclusion criteria were applied in this study. The study by Mikami and colleagues [20] evaluated patients with a wide spectrum of severity (from classes I to V) that required hospitalization due to CAP, and 55% of the patients were categorized as classes IV and V of the PSI score. However, most patients had significant disease severity at presentation as hypoxemia (mean PaO_2 _on presentation = 61 ± 8 mm Hg) and respiratory distress (mean respiratory rate = 29 ± 8 breaths per minute).

### Validation of available studies

We assigned evidence levels to the reviewed studies based on the GRADE system definitions adopted by ATS [17]. Based on these evidence levels, a graded recommendation can be formulated for the use of corticosteroids in severe CAP. Three of the studies were prospective RCT and one was a retrospective cohort study and, as a result, classified low-quality evidence [20], moderate-quality evidence [6], moderate-quality evidence [21], and low-quality evidence [19] as a weak recommendation, a strong recommendation, a weak recommendation, and a strong recommendation, respectively. Therefore, according to the ATS GRADE system, the use of corticosteroids in severe CAP should be graded as a weak recommendation [17].

## Discussion

### Patient selection bias

Selection bias usually is implicated as a plausible explanation for the results observed in clinical trials involving a small patient population. In the present case, the lack of survival benefit observed in the study of Mikami and colleagues [20] may be ascribed to the enrollment of patients with a lower degree of severity in the study (45% of the patients had PSI scores of II or less). However, even though mortality benefits were not observed (and the study was clearly underpowered for mortality), improvements in clinical condition were more frequent in patients treated with corticosteroids.

Nonetheless, the opposite may also be considered. Positive results found by trials with a small number of patients may happen due to either selection bias or chance. In the study by Confalonieri and colleagues [6], a large number of the patients were assessed for eligibility but did not meet the inclusion criteria (69/121, 57%). Moreover, despite the long duration of the inclusion period (3 years) in six centers, only 46 patients were included in the study's final analysis. Also, the number of patients requiring invasive mechanical ventilation (16/23, 70%) in the placebo group was substantially higher than in the hydrocortisone group (7/23, 30%; *P *value = 0.008). Such differences in patient selection may have affected outcomes.

### Safety issues

Several patients admitted to the hospital experience infection-related exacerbations of asthma or chronic obstructive pulmonary disease (COPD) which are severe enough to require the use of systemic corticosteroids for a few days [19,25]. This usually poses a significant dilemma as clinicians caring for these patients often fear that the use of corticosteroids may adversely affect the immune response and, in doing so, compromise the resolution of the infection. While the evaluated studies have not clearly addressed safety issues in a systematic way, at least two studies reported no significant adverse events related to the use of corticosteroids [6,20]. Moreover, in the study by Garcia-Vidal and colleagues [19], a significantly higher number of patients treated with corticosteroids had COPD and their survival rates were superior to those of patients not receiving corticosteroids. The remaining studies [20,21] failed to show increased morbidity or mortality in patients receiving systemic corticosteroids. Therefore, despite the fact that this was not a primary endpoint in any of these studies, it seems that corticosteroid administration is safe in patients receiving antimicrobial therapy.

### Possible explanations for the reported outcomes

#### Duration of corticosteroid therapy

Several studies demonstrated that a short-course high-dose corticosteroid regimen is not associated with any survival benefit and could even be deleterious [26]. In contrast, lower doses for longer periods have been associated with improved survival and earlier shock reversal [7]. The four evaluated studies could be divided according to the duration of corticosteroid therapy in short and long courses. Two studies can be classified as short-course: in the study by Marik and colleagues [21], corticosteroids were given as a single dose, and in the study by Mikami and colleagues [20], they were given once daily for 3 days. In contrast, in the RCT conducted by Confalonieri and colleagues [6], corticosteroids were given by continuous infusion for 7 days, and in the retrospective study of Garcia-Vidal and colleagues [19], they were given (on average) during 11.4 days. A detailed analysis of the results from the four studies demonstrated that those in which corticosteroids were given in a short course showed no benefits concerning the primary endpoints. Conversely, in the studies with longer corticosteroid courses, a significant benefit in mortality and in other clinical variables was registered (Table 2). However, the results from the CORTICUS (Corticosteroid Therapy of Septic Shock) trial [27] showed no survival benefit with a relatively long corticosteroid course (11 days) and challenged this observation. Consequently, the optimum duration of corticosteroid therapy remains controversial.

#### The role of critical illness-related corticosteroid insufficiency

Recent ATS/Infectious Diseases Society of America guidelines [1] state that hypotensive fluid-resuscitated patients with severe CAP should be screened for occult CIRCI (level II evidence). However, among the evaluated studies, only that of Mikami and colleagues [20] analyzed the adrenal function in a subset of patients. While the authors stated that 43% of patients fulfilled criteria for CIRCI, further data on these patients were not reported in the paper. A significant prevalence of CIRCI was described in recent studies [13,14]. Accordingly, Annane and colleagues [28] recently evaluated patients with septic shock (60% of them pneumonia-related) and showed that low doses of corticosteroids were associated with better outcomes in sepsis-associated acute respiratory distress syndrome diagnosed with CIRCI. Therefore, the treatment of undiagnosed underlying CIRCI could have played a role in the improvement of corticosteroid-treated patients. However, recent data suggest that the corticotropin test is unable to identify patients who may respond to steroid therapy [10,27]. For this reason, the usefulness of measuring the adrenal function in patients with severe sepsis remains a contentious issue.

#### Hemodynamic effects of corticosteroids

Currently, the differentiation between severe sepsis and septic shock is made on the basis of vasopressor dependency in the latter group. The results of clinical trials on the use of steroids in septic patients are variable regarding immunomodulation, reversal of organ failures, and impact on the survival rates [7-9,27,29]. In contrast, the effect of hydrocortisone in reducing the need for vasopressors is indisputable [30]. Increase in the mean arterial blood pressure in response to steroids is associated with an increase in systemic resistance and a reduction in cardiac index and heart rate since corticosteroids have a role in regulating the peripheral vascular tone. Such an effect is explained by the inhibition of nitric oxide as well as the increase in the expression of catecholamine receptors. Confalonieri and colleagues [6] report a reduced frequency of 'delayed septic shock' in patients treated with hydrocortisone. Mikami and colleagues [20] also demonstrated a faster stabilization of vital signs in patients treated with corticosteroids but data on mean arterial pressure or lactate levels are not available. Garcia-Vidal and colleagues [19] observed that patients with severe CAP on corticosteroid therapy and equal Charlson score, PSI class, and hypotension at admission do better than those without corticosteroid therapy. However, no further details on clinical data improvement are provided in the article. Hemodynamic benefits associated with steroid therapy could play a significant role in precluding hypoperfusion and multi-organ failure from thus having an impact on mortality.

#### Immunomodulatory effects of corticosteroids

Cytokines and mediators of inflammatory response play a key role in the pathogenesis of severe infections and sepsis-related organ failure and prognosis [31-33]. Several studies demonstrated that the infusion of moderate doses of corticosteroids could blunt the systemic pro-inflammatory cytokine response in severe sepsis [8,9] and the pulmonary inflammation in severe pneumonia [11] and acute lung injury [15]. However, Marik and colleagues [21] were unable to observe any difference in tumor necrosis factor-α course between patients treated with and without hydrocortisone. However, we could speculate that at least part of the clinical improvement observed may be ascribed to the immunomodulatory effects of the steroid infusion, thus hastening the resolution of acute lung injury and organ failures. Of the four studies, two evaluated the effect of corticosteroids on surrogate biomarkers of systemic inflammation and observed more significant reductions in C-reactive protein (CRP) levels in patients treated with corticosteroids [6,20]. Confalonieri and colleagues [6] showed, in patients treated with hydrocortisone, a significant decrease in the multiple organ dysfunction score (MODS) (from 1.2 on day 1 to 0.3 on day 8), whereas in the placebo group MODS remained almost unchanged (from 1.2 to 1.0). Similarly, in the hydrocortisone group, the authors found a significant decrease in CRP, whereas in the placebo group CRP increased even further from day 1 to day 8. Similar findings were observed in gas exchange as demonstrated by the significantly higher PaO_2_/FiO_2 _ratios of patients treated with hydrocortisone on day 8 [6]. The correlation between organ failure, assessed by MODS, and systemic inflammation, assessed by CRP, suggests that a good clinical course is associated with resolution of the inflammatory process whereas a clinical deterioration is associated with ongoing inflammation [33-35]. The exact mechanism of action of corticosteroids contributing to the observed effects is still unknown; however, it is difficult to accept that it results primarily from a direct effect on CRP concentration, decreasing either its rate of synthesis or secretion. If that were the case, patients on steroid therapy would have a blunted acute-phase response with very low levels of CRP in response to an infection [36,37].

## Conclusion

In a small prospective RCT and in a large retrospective cohort study, treatment with systemic corticosteroids showed reductions in mortality or morbidity. However, the small sample sizes [6,20,21], different treatment regimens [6,19-21], and non-experimental design [19] of the studies reviewed may have influenced the results significantly. Different selection criteria may also have resulted in confounding by indication. The concept of using corticosteroids for patients with severe CAP is based on studies that should be categorized as both a weak recommendation (two studies) and a strong recommendation (two studies) with either low- or moderate-quality evidence, thus reflecting a global 'weak recommendation'. Moreover, the results are inconsistent and the mechanism that explains the favorable results remains unclear. Therefore, given the current evidence, it cannot be concluded that corticosteroids should become part of the standard of care for patients admitted to the hospital with severe CAP. However, despite the fact that the rate of side effects from corticosteroids (myopathy, hyperglycemia, and nosocomial infections) was not systematically investigated, all four studies failed to show worse outcomes associated with the use of corticosteroids. Thus, it may be reasonable to conclude that, in patients with COPD or asthma receiving antimicrobial therapy, corticosteroid administration may be considered safe. Particularly considering the recent results of trials of corticosteroids in septic shock [27], future RCTs should overcome the methodological flaws in the designs of the currently available studies. To determine the risks and benefits of adding corticosteroids to the treatment of patients with severe CAP, large prospective randomized studies are necessary.

## Key messages

• Few studies have evaluated the use of systemic corticosteroids in patients with severe community-acquired pneumonia (CAP). On the basis of their results, the use of corticosteroids as adjunctive therapy in severe CAP should be categorized as a weak recommendation (two studies) and a strong recommendation (two studies) with either low- or moderate-quality evidence.

• No evidence of adverse outcomes or harm is present in the studies that evaluated the use of systemic corticosteroids in patients with severe CAP. Thus, in patients with chronic obstructive pulmonary disease or asthma receiving antimicrobial therapy for severe CAP, concomitant administration of moderate doses of systemic corticosteroids may be considered safe.

• In view of the recent results of the CORTICUS (Corticosteroid Therapy of Septic Shock) study, which evaluated the use of corticosteroids in septic shock, future randomized controlled trials should overcome the methodological flaws in the designs of the currently available studies. To determine the risks and benefits of adding corticosteroids to the treatment of patients with severe CAP, large prospective randomized studies involving a more homogeneous group of patients are necessary.

## Abbreviations

ATS = American Thoracic Society; CAP = community-acquired pneumonia; CIRCI = critical illness-related corticosteroid insufficiency; COPD = chronic obstructive pulmonary disease; CRP = C-reactive protein; FiO_2 _= fraction of inspired oxygen; LOS = length of stay; MODS = multiple organ dysfunction score; PaO_2 _= arterial partial pressure of oxygen; PSI = pneumonia severity index; RCT = randomized controlled trial.

## Competing interests

The authors declare that they have no competing interests.

## Authors' contributions

JIFS, PP, and MS contributed to the study conception and design, carried out and participated in data analysis, and drafted the manuscript. FAB, PTB, and HCFN conceived the study, participated in its design and coordination, supervised data analysis, and helped to draft the manuscript. All authors read and approved the final manuscript.
